# Hot Phases Cardiomyopathy: Pathophysiology, Diagnostic Challenges, and Emerging Therapies

**DOI:** 10.1007/s11886-024-02168-6

**Published:** 2025-01-09

**Authors:** Giulia Bassetto, Federico Angriman, Carola Pio Loco detto Gava, Alessia Paldino, Maria Perotto, Luca Bordignon, Marta Gigli, Matteo Dal Ferro, Laura Massa, Alessandro Altinier, Antonio De Luca, Gianfranco Sinagra, Marco Merlo

**Affiliations:** Center for Diagnosis and Treatment of Cardiomyopathies, Cardiovascular Department, Azienda Sanitaria Universitaria Giuliano Isontina (ASUGI), European Reference Network for Rare, University of Trieste, Via P. Valdoni 7, 34100 Trieste, Italy

**Keywords:** Hot-phases, Cardiomyopathy, Myocarditis, Inflammation, Desmoplakin, Arrhythmic risk

## Abstract

**Purpose of Review:**

Hot phases are a challenging clinical presentation in arrhythmogenic cardiomyopathy (ACM), marked by acute chest pain and elevated cardiac troponins in the absence of obstructive coronary disease. These episodes manifest as myocarditis and primarily affect young patients, contributing to a heightened risk of life-threatening arrhythmias and potential disease progression. This review aims to synthesize recent research on the pathophysiology, diagnostic challenges, and therapeutic management of hot phases in ACM.

**Recent Findings:**

Hot phases have been linked to genetic mutations, particularly in desmosomal proteins such as Desmoplakin (*DSP*). Diagnostic challenges include differentiating hot phases from isolated acute myocarditis, through identification of red flags and a multimodal approach, including CMR, FDG-PET, endomyocardial biopsy and genetic testing. Emerging therapies, such as immunosuppressive and anti-inflammatory treatments, show promise in managing hot-phase episodes.

**Summary:**

Hot phases in ACM present a significant risk for arrhythmias and disease progression, necessitating a comprehensive diagnostic and therapeutic management. A multimodal diagnostic approach is essential for accurate diagnosis, but further research is needed to refine these strategies and improve prognosis for affected patients.

## Introduction

Myocarditis is an inflammatory disease of the myocardium with a wide spectrum of clinical presentations, ranging from chest pain without coronary obstruction to ventricular arrhythmias and even cardiogenic shock.[1] This condition can progress to dilated cardiomyopathy (DCM) in up to 30% of cases.[2, 3] In the last years, a complex interplay between genetics and myocarditis has emerged. Particularly, these two elements seem to overlap in the case of arrhythmogenic cardiomyopathy (ACM), a disease characterized by progressive fibro-fatty replacement of the myocardium that can affect the right (Arrhythmogenic Right Ventricular Cardiomyopathy -ARVC), the left (Arrhythmogenic left ventricular arrhythmogenic—ALVC) or both ventricles (biventricular ACM). [4] ACM predisposes patients to life-threatening ventricular arrhythmias, sudden cardiac death (SCD) and heart failure.[5] As recently highlighted, ACM can also present with chest pain and elevation of cardiac troponins in the absence of obstructive coronary disease: a condition defined as “Hot phases”. [6, 7] These episodes affect predominantly young patients and the differential diagnosis with classic acute myocarditis is a challenge. These events are particularly significant as they have been associated with acute worsening of the disease and significant increase in the risk of ventricular arrhythmias. [8].

This review aims to synthesize the latest research on “Hot phases”, exploring their proposed pathophysiological basis alongside their clinical implications, diagnostic challenges, and emerging therapeutic targets.

## Pathophysiology of Hot Phases in Arrhythmogenic Cardiomyopathy

Increasing body of research suggests that the pathophysiology of ACM involves a complex interplay of genetic, inflammatory, and immune system backgrounds. “Hot phases” are characterized by myocarditis episodes that might be triggered by various factors, including exercise, viral infections, and autoimmune mechanisms.

### Genetic Background

A significant role of genetics in acute myocarditis and inflammatory cardiomyopathies has been suggested by several studies: in a multicentric study, genetic variants in cardiomyopathy-related genes were described in 8% of acute myocarditis patients compared to < 1% of healthy controls. [9] Furthermore, a high prevalence of titin truncating variants (*TTNtv*) was found in patients with EMB-confirmed lymphocytic myocarditis [10] and DMD mutated patients seem to develop heart failure more frequently when inflammation is present on CMR.[11] Additionally, mutations in *FLNC*, *BAG3* and *RBM20*, all linked to ACM, appear to be associated with severe forms of acute myocarditis.[12].

Moreover, some reports documented the presence of homozygous or compound heterozygous mutations in cardiomyopathy-related genes in unrelated paediatric patients presenting with acute myocarditis.[13].

In “Hot phase” patients the most commonly mutated gene is Desmoplakin (*DSP*). [14] *DSP* is the most abundant desmosomal protein in the heart and the skin, and it anchors the desmosomes to intermediate filaments. As described by Smith et al., *DSP* mutations are associated with a predominant LV involvement with systolic dysfunction, subepicardial fibrosis and inflammatory hot phases, usually preceding LV dysfunction. This phenotype differs from the classic ARVC form linked to *PKP2 variants* [15].

Interestingly, the presence of *DSP* variants in patients with acute myocarditis was reported to be associated with worse outcomes in acute myocarditis patients, as DSP mutation carriers had a higher risk of ventricular arrhythmias and recurrent myocarditis. [16].

Although less common, genetic variants of *PKP2* and *DSG2* were also respectively detected in up to 21% and 13% of “Hot phase” cases in some reports. [17].

However, a clear mechanism linking ACM-related gene variants to “Hot phases” has still to be outlined. ACM is primarily associated with mutations in genes encoding five desmosomal proteins, including the aforementioned plakophilin-2 (*PKP2*), desmoplakin (*DSP*), junctional plakoglobin (*JUP*), desmocollin-2 (*DSC2*) and desmoglein (*DG*). The desmosome is a multiprotein complex belonging to intercellular junctions that allows myocyte adhesion and electrical coupling. Mutations in desmosomal genes disrupt the integrity of the intercalated discs, leading to myocardial cell detachment, apoptosis, and subsequent fibro-fatty replacement. [18].

Recent studies have also highlighted the role in ACM of non-desmosomal genes, such as filamin C (*FLNC*)[19, 20] and phospholamban (*PLN*)[21], that encode proteins involved in calcium handling and the sarcomere.

### Inflammation and Immune Response

Inflammation has an important and still controversial role in the physiopathology of cardiomyopathies, and even more so in ACM “Hot phases”. Histopathological studies have revealed high prevalence of inflammatory infiltrates, along with apoptotic myocytes, in ARVC hearts and their association with more severe biventricular involvement. [22] Whether the inflammation represents a trigger for the expression of a genetic background, or only represents an epiphenomenon in a susceptible patient is still unknown. The definition of the exact role of these processes would be of uttermost importance to direct the management and therapy of “hot phases” cardiomyopathies.

In murine models, an inflammatory phase, including necrotic foci and inflammatory infiltrates, seems to precede the onset of the overt disease weeks before developing symptoms and ventricular dysfunction. [23] This finding reinforces the hypothesis that “hot phases” might contribute to pathogenesis and disease progression in ACM through myocyte degeneration and inflammatory response that mimic acute myocarditis.

Multiple theories have been proposed on the interplay of genetics and inflammation in ACM and “Hot phases”: it has been suggested that genetically determined desmosomal dysfunction could induce myocyte apoptosis and subsequent ventricle inflammation. [24] Another hypothesis is that viral infections might cause myocardial inflammation and apoptosis, followed by desmosomal dysfunction. [25].

It is possible that a “second-hit theory” model, already proposed in DCM, could be applied to ACM “Hot phases” [26]. In this case, some genetic defects may favor myocardial susceptibility to inflammatory insults, such as viral infections, causing myocardial damage and fibrofatty replacement. According to this theory, desmosomal mutations cause downregulation of the *Wnt/B-catenin* cascade and activation of the *Hippo/YAP pathway*, upregulating the expression of pro-apoptotic genes and weakening intercellular junctions: exogenous agents can then induce myocardial damage with endogenous Damage-Associated Molecular Patterns (DAMPs) release, activation of the immune system and inflammatory-mediated myocyte damage, creating a vicious circle.[27].

The immune response during “Hot phases” is characterized by both innate and adaptive immune mechanisms: and T-lymphocytes, macrophages and neutrophils are the most commonly found cells in ACM hearts. [28, 29].

In the early phases of the disease, damaged cardiomyocytes are cleared by myeloperoxidase secreting neutrophils (MPO +), which are progressively substituted by M2 macrophages. The release of pro-inflammatory cytokines and chemokines, such as IL-1, IL-6, TNFapha and IFN gamma, not only damages the myocardium, but also recruits additional immune cells and activates adaptive immunity, creating a vicious cycle of inflammation and tissue injury. [30].

Complement activation in “Hot phases” is currently under study: high levels of C3, C5 and C9 in the serum of ACM patients and an improvement in cardiac function in DES mutated mice after C5a receptors inhibition have been demonstrated. [31, 32].

Some cytokines are also involved in “Hot phases”. For instance, IL-1β production has been observed in *Dsg2mut/mut* mice cardiomyocytes. NF-KB, a nuclear transcriptional factor involved in IL-1B production, is overexpressed both in *Dsg2mut/mut* animal models and cardiomyocytes derived from human induced pluripotent stems (hiPSC-CMs) carrying a *PKP2* mutation. [26]. Additionally, through NF-KB overexpression, *PKP2* mutated hiPSC-CMs can secrete inflammatory cytokines themselves, in the absence of immune cells.[33].

This inflammatory *milieu*, also through the development of fibrosis, can destabilize the myocardial substrate, increasing the propensity for arrhythmias.

In a recent study*, DSG2Mut* hiPSC-CMs presented NF-KB upregulation associated with electrophysiological abnormalities and *DSG2* suppression via siRNA showed partial normalization of these electrophysiologic changes.[34]Moreover, Chelko et al. observed that in *Dsg2 mut/mut* mice NF-KB signalling in cardiomyocytes attracts C–C motif chemokine receptor-2 positive macrophages (CCR2 + cells) where they mediate myocardial injury, contractile dysfunction and arrhythmias. [33].

### Role of Viral Infections and Autoimmunity

As previously reported, inflammatory infiltrates have been described in autoptic series of ARVC patients, suggesting that, similarly to DCM, ACM could be triggered by myocarditis which, in western countries, is most commonly related to viral infections or autoimmune damage.

Cardiotropic virus sequences (such as Coxsackie virus B3, adenovirus CMV and Parvovirus) have been detected in the hearts of patients with ARVC and no family history of cardiomyopathy, so that, initially, a role of infectious agents was proposed in sporadic cases. [35] Nevertheless, current evidence suggests that viruses could be innocent bystanders in a cardiomyopathic environment, rather than causal agents of the disease. [36].

Conversely, it has been proposed that the inflammatory response might be an autoimmune reaction triggered by the exposure of cryptic myocardial antigens following myocyte injury.

Cytotoxic T cells can contribute to cardiomyocyte damage in ACM in the absence of viral infections. [37] Also, autoantibodies against cardiac proteins (AHA and AIDA) have been detected in patients’ serum and seemed to be associated with disease severity in ARVC patients, but larger studies are needed to confirm this hypothesis. [38, 39].

## Clinical Manifestations

“Hot phases” usually affect young patients and can be the first clinical manifestation of ACM in paediatric cohorts. [7].

Patients present with acute chest pain and/or cardiac enzyme release without significant coronary artery disease, compatible with infarct-like acute myocarditis. Therefore, patients can present relapsing episodes of chest pain or chronic asymptomatic release of troponin.

Hemodynamic instability, although rare, is possible. Cases of cardiogenic shock were described[40], as well as severe bradyarrhythmias. [41].

SCD has been reported in up to 9% of patients included in recent case series [16], and arrhythmic instability due to life-threatening tachyarrhythmias is described during “Hot phase” episodes. [42, 43].

Chest pain can be associated with ST changes on EKG. In this setting, electrocardiographic abnormalities suggestive of a cardiomyopathic background include low and fragmented QRS voltages on peripheral leads and negative T waves in inferolateral leads. However, EKG can be normal at first presentation. [7].

Currently, “Hot phases” remain a challenge for the physician, as they can occur unpredictably, in a multitude of clinical settings and do not necessarily fit in the classical progression of ACM.

## Diagnostic Challenges

Recognizing “Hot phase” cardiomyopathy and differentiating it from common myocarditis poses significant challenges. Indeed, due to the current absence of a reliable and standardized diagnostic criteria and work-up, it is essential to approach these cases with a “cardiomyopathy mindset”, aimed at recognizing the red flags of a possible more complex underling condition (Table 1).

**Table 1:** Clinical and instrumental “Red flags” of “Hot phases” cardiomyopathy

RED FLAGS	
Clinical History	• Recurrent episodes of chest pain • Family history of SCD or CMP • Chronic release of Troponin
EKG	• Low QRS voltages • Fragmented QRS • Inferolateral T-wave inversion • Persistent ventricular arrhythmias
Imaging	• Persistent LV dysfunction • Ring-like LGE pattern at CMR

Key: *CMP* cardiomyopathy, *CMR* cardiac magnetic resonance, *LGE* late gadolinium enhancement, *LV* left ventricular, *SCD* sudden cardiac death

A positive family history of cardiomyopathy, sudden cardiac death or myocarditis, alongside relapsing episodes of chest pain, should be investigated by the physician to reach an accurate diagnosis. [44].

**Fig. 1:**
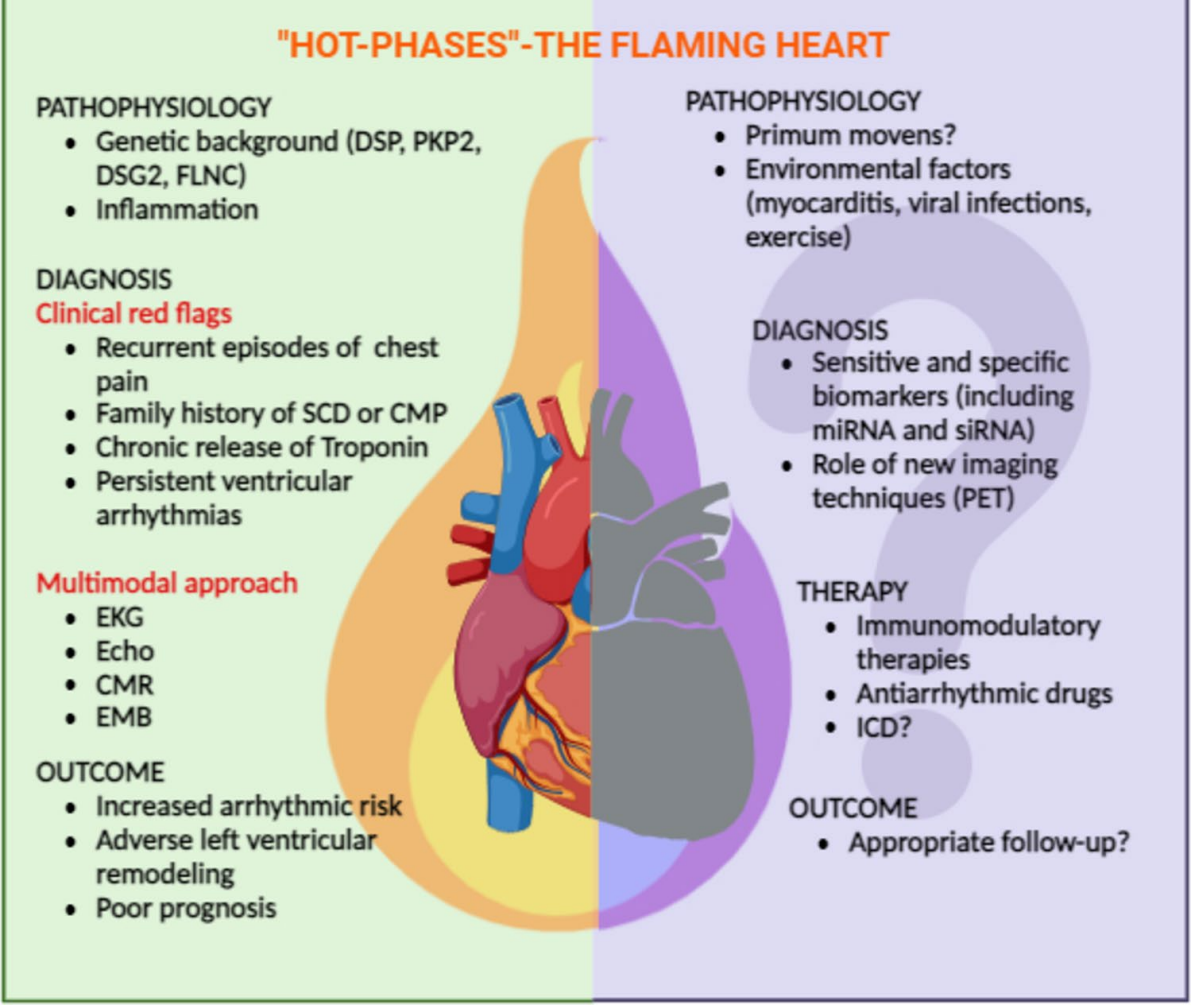
Current management and standing questions in “Hot phases” cardiomyopathy. Key: *CMP* cardiomyopathy, *CMR* cardiac magnetic resonance, *DSG2* desmoglein-2, *DSP* desmoplakin, *EMB* endomyocardial biopsy, *EKG* electrocardiogram, *FLNC* filamin C, *ICD* implantable cardioverter-defibrillator, *PET* positron emission tomography, *PKP2* plakophilin-2, *SCD* sudden cardiac death, *siRNA* Short interfering RNA

However, there is a growing need for novel diagnostic tools that can detect myocardial inflammation and predict arrhythmic risk during “Hot phases”. Multimodal diagnostic workup, including EKG, echocardiogram, CMR, nuclear imaging and, when necessary, invasive assessment with EMB and electrophysiological studies, must be considered for appropriate management of these complex patients (Fig. 1).

In such uncharacterized conditions, referral centres have a role not only for the management of single patients but also in building a solid data collection. An appropriate use of tertiary centres would be essential to unveil the many questions that still surround “Hot phase”.

### Biomarkers

Except for acute and chronic release of cardiac enzymes, the search for reliable biomarkers that can detect or predict “hot phase” in ACM is ongoing. Elevated levels of inflammatory markers such as C-reactive protein (CRP) have been reported during “Hot phase”, although not frequently. Even though CRP is non-specific and cannot distinguish ACM from other inflammatory or ischemic cardiac conditions, it currently represents, alongside troponin, the available biomarker in this clinical context. Overexpression of inflammatory cytokines and chemokines in ACM, including IL-1β, IL-6, INFγ and tumour necrosis factor-α (TNFα) have been described in animal models, but studies assessing their roles as biomarkers of “hot phase”are warranted. [45].

Similarly elevated soluble suppression of tumorigenicity-2 (sST2), growth differentiation factor-15 (GDF-15) and NT-proBNP may predict biventricular ACM, but no data are available in the context of “Hot phase” cardiomyopathy. [46].

Research into more specific biomarkers, such as microRNAs and autoantibodies, is needed to improve diagnostic accuracy and guide treatment during “Hot phases” [47].

### Imaging Modalities

Cardiac magnetic resonance imaging (CMR) has emerged as a critical tool in the diagnosis of “Hot phase” cardiomyopathy in the context of ACM and non-dilated left ventricular cardiomyopathy (NDLVC). CMR can detect edema, and fibrosis with high sensitivity and specificity. The use of late gadolinium enhancement (LGE) allows for the visualization of myocardial scarring. The presence of non-ischaemic LGE has been described in up to 90% of “Hot phase” patients, particularly affecting the subepicardial inferolateral and septal regions. [7] A circumferential subepicardial pattern, called “ring-like”, is most typical and associated with *DSP* and *FLNC* mutations. [15].

Additionally, T1 and T2 mapping techniques could provide valuable insights into the extent and activity of the disease during “Hot phases”, showing an increase in T1 and T2 relaxation times in the presence of myocardial damage and edema.

Positron emission tomography (PET) imaging is useful in the diagnosis of cardiac sarcoidosis and acute myocarditis and represents a promising tool to assess myocardial inflammation in ACM, as up to 36% can present LV myocardial uptake at PET. [48]This imaging modality is mostly helpful when CMR is not feasible (i.e. when an implantable device is present).[49].

Considering that active inflammation could be linked to an increased arrhythmic risk, this technique may be particularly useful in identifying patients at risk of arrhythmic events during hot phases, although its routine use in clinical practice is still under investigation.

### Endomyocardial Biopsy

Differential diagnosis between cardiac sarcoidosis and acute myocarditis might be difficult even after a complete non-invasive workup, especially in cases where lungs are not involved. In these cases, performing an endomyocardial biopsy (EMB) with histological and immune-histochemical analysis is mandatory. [50].

Myocardial inflammation has been reported in up to 75% of hearts at autopsy, particularly in *DSP*-related ARVC. [51].

However, highlighting once more the ongoing debate on “Hot-phases” pathogenesis, a recent case series found that apoptosis rather than active inflammation can be found on EMB of “Hot-phase” patients with P/LP mutations in cardiomyopathy-related genes. [52].

Although false negatives can be present, especially when sampling from the RV, electroanatomic-voltage mapping (EAVM)-guided EMB, by highlighting areas of EP abnormalities, has shown excellent results in diagnosing active myocardial inflammation. [53].

Apart from allowing to rule out cardiac sarcoidosis and viral myocarditis, the detection of active inflammation poses the question of whether to start immunosuppressive therapy.

### Role of Genetic Testing and Familial Screening

In acute myocarditis patients, the risk of an associated inherited cardiac disease is unclear and AM is not mentioned in the guidelines as a criterion to suggest genetic testing.[54].

Performing genetic testing in patients with acute myocarditis and a familial background of cardiomyopathy or SCD was effective in identifying desmosomal mutations and family screening allowed to diagnose an ALVC phenotype in more than a third of mutation carriers. [44].

These results outline the importance of identifying red flags for performing genetic testing in patients with AM that present features of clinical complexity, such as relapsing episodes of myocarditis, chronic troponin release, family history of cardiomyopathy, ring-like pattern on CMR, persistent severe left ventricular dysfunction, defined as LV dysfunction which does not respond as predicted to optimized medical therapy, or persistent sustained ventricular arrhythmias, defined by the need for multiple medical therapy adaptation with poor arrhythmic burden reduction; also, possibly, the presence of apoptosis on EMB (Table 1).

Whether genetic testing should be performed systematically in AM patients is currently under debate and multicentric studies are needed to shed light on this theme, but assessing family history in this subset of patients is of utmost importance.

As for familial screening, first-grade family members should undergo genetic testing. In case of identification of the same variant is identified in an asymptomatic relative, CMR should be performed, even when echocardiography and ECG are normal. [54].

## Therapeutic Approaches and Lifestyle Modifications

The management of ACM during “Hot phases” requires a multifaceted approach that addresses both the arrhythmic risk, the underlying inflammatory process and, in certain cases, ventricular dysfunction.

Given the possible central role of inflammation in the pathophysiology of “Hot phases”, there is growing interest in the use of immunosuppressive and anti-inflammatory therapies in this field.

Hot phases have not yet been object of clinical randomized trials or large observational studies, and currently used regimes of immunosuppressive therapies have been adapted from Acute Myocarditis guidelines[55]. In the setting of autoimmune myocarditis and virus-negative myocarditis, immunosuppressive is recommended in cases refractory to optimized medical therapy, and long term follow up of TIMIC trial showed the efficacy of prednisone and azathioprine combination in chronic inflammatory cardiomyopathies. [56].

Concerning genetic cardiomyopathies presenting myocardial inflammation, a pilot report on the use of immunosuppression showed a reduction of myocardial inflammation and a relative decrease of adverse events while on treatment, without reporting serious adverse events. [57] These results certainly call for further investigation to prove the efficacy of immunosuppressive therapy in this setting.

Most importantly the choice for initiation of IMT should be tailored to the single patient and should be managed by a multidisciplinary Cardioimmunology team.[58] The presence of symptoms, systolic dysfunction, persistent troponin release, ventricular arrhythmias, LGE progression at CMR should be considered at the beginning of the therapy and during follow-up to guide optimal treatment.

This aspect needs to be the focus of future research in hot phases cardiomyopathy.

Recent studies have explored the use of targeted immunomodulatory therapies, inhibiting GSK3 β [59, 60], the NFκB cascade [61] and CCR2 macrophages on ACM animal models. [62].

Colchicine use is currently understudy in chronic inflammatory cardiomyopathy patients (CMP-MYTHiC), accounting also ACM patients with documented “Hot phases” [63]. This aspect needs to be the focus of future research in “hot phases” cardiomyopathy.

Nevertheless, “Hot phases” patients with P/LP mutations in cardiomyopathy-related genes and reduced ejection fraction showed reverse remodelling with anti-neurohormonal therapy when apoptosis, rather than significant inflammation, was present on EMB. [52].

As for exercise restrictions, strenuous exercise has been linked to progression of ACM and its restriction has shown a reduction in ventricular arrhythmic burden in ACM patients carrying an ICD. [64] Similarly, limitation of physical activity in acute myocarditis patients is recommended from 3 to 6 months from the acute events. [54] Data on the impact of exercise on “Hot phases” are not yet available. As a general recommendation, patients are often advised to avoid competitive sports and strenuous exercise, which could exacerbate myocardial inflammation and increase arrhythmic risk.

## Prognosis and Long-Term Outcomes

As previously stated, desmosomal mutations in acute myocarditis are reported to be associated with a worse prognosis in terms of arrhythmic events.

While adverse prognostic factors in ACM have been identified, knowledge on risk stratification in “Hot phase” cardiomyopathy is limited. Current data are not sufficient to assess the prognosis of patients with “Hot phases” ACM which could depend on several factors, including the extent of myocardial involvement, the frequency and severity of arrhythmic events, and the effectiveness of therapeutic interventions.

A recent study reported a higher prevalence of life-threatening arrhythmias in DSP cardiomyopathy patients in case of severe LV dysfunction and RV dysfunction, but episodes of AM did not predict a higher arrhythmic risk.[65] On the other hand, “Hot phases” were found to be a negative prognostic factor for heart failure related outcomes. [7].

It is imperative to assess the appropriate management of these patients, including antiarrhythmic therapy, lifestyle modifications, and ICD implantation, through multi-centric prospective studies.

## Future Directions and Open Problems

The understanding of “Hot phases” in cardiomyopathies (mostly ACM) is still evolving, and several areas of research need to be investigated to improve diagnosis, risk stratification, and treatment. (Fig. 1) Future studies should focus on:

1. Elucidating the molecular mechanisms of inflammation: a deeper understanding of the molecular pathways that drive inflammation during “Hot phases” could lead to the development of targeted therapies that prevent or mitigate these episodes.
2. Identifying specific biomarkers: discovery of specific biomarkers that can predict or detect “Hot phases” would greatly enhance the ability to diagnose and manage them more effectively.
3. Assessing the role of novel imaging techniques: advances in imaging modalities, such as PET and CMR, should be further explored to determine their utility in detecting and monitoring myocardial inflammation and fibrosis during “Hot phases”.
4. Evaluating new therapeutic strategies: clinical trials investigating the efficacy of novel immunomodulatory and anti-inflammatory agents in ACM and “Hot phases” cardiomyopathy are needed to establish new treatment paradigms for managing “hot phases”
5. Understanding the impact of exercise: more research is required to define the optimal exercise recommendations for patients with ACM, particularly in the context of preventing “Hot phases” and reducing arrhythmic risk.

## Conclusions

“Hot phases cardiomyopathy” is a newly emerged clinical entity in the complex and uncharacterized overlap of myocarditis, ACM, DCM and NDLVC. This condition surely represents a significant challenge in current clinical practice. “Hot phases” episodes exacerbate the arrhythmic risk and might contribute to disease progression. Recent research has advanced our understanding of the pathophysiological mechanisms underlying hot phases, but it has also highlighted the importance of a multimodal diagnostic approach and the need for effective therapeutical strategies. However, much remains to be learned, and ongoing research is essential to improve the prognosis and quality of life for patients with “Hot phases”. It is once more to be stressed, in the current healthcare landscape, that third-level centres, via multicentre cooperation, should represent the heart of research activities and data collection.

## Key References

- Bariani R, Cipriani A, Rizzo S, Celeghin R, Bueno Marinas M, Giorgi B, et al. ‘Hot phase’ clinical presentation in arrhythmogenic cardiomyopathy. Eur Pacing 2021 Jun 7;23(6):907–17.
  - This study provides an extensive phenotypic characterization of patients presenting with “hot-phases.”
- Wang W, Murray B, Tichnell C, Gilotra NA, Zimmerman SL, Gasperetti A, et al. Clinical characteristics and risk stratification of desmoplakin cardiomyopathy. EP Eur. 2022 Feb 1;24(2):268–77.
  - This paper focuses on the prognostic impact of “hot phases” in ACM, showing a higher risk of developing arrhythmias and heart failure.
- Ammirati E, Raimondi F, Piriou N, Sardo Infirri L, Mohiddin SA, Mazzanti A, et al. Acute Myocarditis Associated With Desmosomal Gene Variants. JACC Heart Fail. 2022 Oct;10(10):714–27.
  - This study showed the significance of genotyping patients with acute myocarditis and red flags for cardiomyopathies, as patients with desmosomal variants have a higher risk of experiencing life-threatening arrhythmias.
- Table 2 shows the most important cohort data contributions on “hotphases”

**Table 2:** Most representative cohort studies on “Hot-phases”

Reference	Population	Main results	Conclusions
Lopez Ayala, Hearth Rhythm 2015[66]	131 ARVC patients 47 ALVC pateints 64 nonaffected mutation carrying relatives	Seven patients presented AM, preceding LV dysfunction in 2 and VTs in 2. Am clustered in families carrying a DSP truncating variant	Hot phases can cause progression of ACM and should be suspected if myocarditis is associated to family history of ACM
Piriou, ESC Heart Failure 2020[44]	6 families with at least one patient with acute myocarditis and one with cardiomyopathy or SCD	- 5 probands carried a DSP mutation + - 39% of DSP family members had ACM	Genetic testing should be considered in patients with AM and family history for cardiomyopathy/SCD
Smith, Circulation 2020[15]	107 patients with DSP mutation vs 81 PKP2 carriers	15% of DSP mutation carriers showed AM with left ventricular LGE at CMR and preserved LVEF	DSP cardiomyopathy presents episodes of AM with LV fibrosis preceding LV dysfunction
Bariani, Europace 2021[7]	23 patients with ACM experiencing hot phases	- Mean age at onset 24 years - Genetic testing positive in 77% - DSP most involved gene	Hot-phases are a clinical presentation of ACM that affects pediatric patients and DSP carriers. Multiparametric characterization is essential
Scheel, JACC 2021[14]	12 patients with AM as initial ACM presentation	- All females - DSP enrichment - LV involvement at CMR - All developed ACM by Task Force Criteria 2010	ACM can initially present as AM, affecting females, carrying DSP mutations and showing LV involvement
Wang, EP Eur 2022[8]	91 individuals with DSP mutations	- 22% of patients presented hot-phases - Hot phases associated with MVA and heart failure	Hot-phases in DSP carriers are associated with worse disease outcomes
Ammirati, JACC HF 2022[16]	97 patients with acute myocarditis	AM with desmosomal mutations had higher risk of cardiac adverse events compared to negative genetic testing and those who did not perform it (62.3% vs 17.5% vs 5.3% at 5 years)	Patients with AM and desmosmal variants have a higher risk of MVA and myocarditis recurrences

## Data Availability

No datasets were generated or analysed during the current study.

## References

[1] Caforio ALP, Pankuweit S, Arbustini E, Basso C, Gimeno-Blanes J, Felix SB, et al. Current state of knowledge on aetiology, diagnosis, management, and therapy of myocarditis: a position statement of the European Society of Cardiology Working Group on Myocardial and Pericardial Diseases. Eur Heart J. 2013;34(33):2636–48. 23824828 10.1093/eurheartj/eht210

[2] Management of Acute Myocarditis and Chronic Inflammatory Cardiomyopathy | Circulation: Heart Failure [Internet]. [cited 2024 Aug 31]. Available from: https://www.ahajournals.org/doi/10.1161/CIRCHEARTFAILURE.120.007405 10.1161/CIRCHEARTFAILURE.120.007405 PMC7673642 33176455

[3] Merlo M, Ammirati E, Gentile P, Artico J, Cannatà A, Finocchiaro G, et al. Persistent left ventricular dysfunction after acute lymphocytic myocarditis: Frequency and predictors. PLoS ONE. 2019;14(3):e0214616. 30921422 10.1371/journal.pone.0214616 PMC6438511

[4] Corrado D, PerazzoloMarra M, Zorzi A, Beffagna G, Cipriani A, Lazzari MD, et al. Diagnosis of arrhythmogenic cardiomyopathy: The Padua criteria. Int J Cardiol. 2020;15(319):106–14. 10.1016/j.ijcard.2020.06.005 32561223

[5] Gilotra NA, Bhonsale A, James CA, te Riele ASJ, Murray B, Tichnell C, et al. Heart Failure Is Common and Under-Recognized in Patients With Arrhythmogenic Right Ventricular Cardiomyopathy/Dysplasia. Circ Heart Fail. 2017;10(9):e003819. 28874384 10.1161/CIRCHEARTFAILURE.116.003819

[6] Sen-Chowdhry S, Syrris P, Prasad SK, Hughes SE, Merrifield R, Ward D, et al. Left-dominant arrhythmogenic cardiomyopathy: an under-recognized clinical entity. J Am Coll Cardiol. 2008;52(25):2175–87. 19095136 10.1016/j.jacc.2008.09.019

[7] Bariani R, Cipriani A, Rizzo S, Celeghin R, Bueno Marinas M, Giorgi B, et al. ‘Hot phase’ clinical presentation in arrhythmogenic cardiomyopathy. Eur Eur Pacing Arrhythm Card Electrophysiol J Work Groups Card Pacing Arrhythm Card Cell Electrophysiol Eur Soc Cardiol. 2021;23(6):907–17. 10.1093/europace/euaa343 PMC8184227 33313835

[8] Wang W, Murray B, Tichnell C, Gilotra NA, Zimmerman SL, Gasperetti A, et al. Clinical characteristics and risk stratification of desmoplakin cardiomyopathy. EP Eur. 2022;24(2):268–77. 10.1093/europace/euab183 PMC8824516 34352074

[9] Lota AS, Hazebroek MR, Theotokis P, Wassall R, Salmi S, Halliday BP, et al. Genetic Architecture of Acute Myocarditis and the Overlap With Inherited Cardiomyopathy. Circulation. 2022;146(15):1123–34. 36154167 10.1161/CIRCULATIONAHA.121.058457 PMC9555763

[10] Artico J, Merlo M, Delcaro G, Cannat à A, Gentile P, De AG, et al. Lymphocytic Myocarditis. J Am Coll Cardiol. 2020;75(24):3098–100. 32553263 10.1016/j.jacc.2020.04.048

[11] Mavrogeni S, Papavasiliou A, Spargias K, Constandoulakis P, Papadopoulos G, Karanasios E, et al. Myocardial inflammation in duchenne muscular dystrophy as a precipitating factor for heart failure: a prospective study. BMC Neurol. 2010;10(1):33. 20492678 10.1186/1471-2377-10-33 PMC2885327

[12] Tiron C, Campuzano O, Fernández-Falgueras A, Alcalde M, Loma-Osorio P, Zamora E, et al. Prevalence of pathogenic variants in cardiomyopathy-associated genes in myocarditis. Circ Genomic Precis Med. 2022;15(3):e003408. 10.1161/CIRCGEN.121.003408 35522179

[13] Belkaya S, Kontorovich AR, Byun M, Mulero-Navarro S, Bajolle F, Cobat A, et al. Autosomal recessive cardiomyopathy presenting as acute myocarditis. J Am Coll Cardiol. 2017;69(13):1653–65. 28359509 10.1016/j.jacc.2017.01.043 PMC5551973

[14] Scheel PJ, Murray B, Tichnell C, James CA, Tandri H, Calkins H, et al. arrhythmogenic right ventricular cardiomyopathy presenting as clinical myocarditis in women. Am J Cardiol. 2021;15(145):128–34. 10.1016/j.amjcard.2020.12.090 33460606

[15] Smith ED, Lakdawala NK, Papoutsidakis N, Aubert G, Mazzanti A, McCanta AC, et al. Desmoplakin cardiomyopathy, a fibrotic and inflammatory form of cardiomyopathy distinct from typical dilated or arrhythmogenic right ventricular cardiomyopathy. Circulation. 2020;141(23):1872–84. 32372669 10.1161/CIRCULATIONAHA.119.044934 PMC7286080

[16] Ammirati E, Raimondi F, Piriou N, SardoInfirri L, Mohiddin SA, Mazzanti A, et al. Acute myocarditis associated with desmosomal gene variants. JACC Heart Fail. 2022;10(10):714–27. 36175056 10.1016/j.jchf.2022.06.013

[17] Bariani R, Rigato I, Cipriani A, Bueno Marinas M, Celeghin R, Basso C, et al. Myocarditis-like episodes in patients with arrhythmogenic cardiomyopathy: a systematic review on the so-called hot-phase of the disease. Biomolecules. 2022;12(9):1324. 36139162 10.3390/biom12091324 PMC9496041

[18] Calore M, Lorenzon A, De Bortoli M, Poloni G, Rampazzo A. Arrhythmogenic cardiomyopathy: a disease of intercalated discs. Cell Tissue Res. 2015;360(3):491–500. 25344329 10.1007/s00441-014-2015-5

[19] Gigli M, Stolfo D, Graw SL, Merlo M, Gregorio C, Nee Chen S, et al. Phenotypic expression, natural history, and risk stratification of cardiomyopathy caused by filamin C truncating variants. Circulation. 2021;144(20):1600–11. 34587765 10.1161/CIRCULATIONAHA.121.053521 PMC8595845

[20] Vrettos A, Demetriades P, Ortiz M, Domínguez F, García-Pavía P, Suárez-Mier MP, et al. Pathogenic truncating filamin C mutations presenting as acute myocarditis: a case series with insights from cardiac magnetic resonance and histological analysis. Eur Heart J - Case Rep. 2024;8(3):ytae111. 38476289 10.1093/ehjcr/ytae111 PMC10928485

[21] Vafiadaki E, Glijnis PC, Doevendans PA, Kranias EG, Sanoudou D. Phospholamban R14del disease: The past, the present and the future. Front Cardiovasc Med [Internet]. 2023 Apr 18 [cited 2024 Aug 31];10. Available from: https://www.frontiersin.org/journals/cardiovascular-medicine/articles/10.3389/fcvm.2023.1162205/full 10.3389/fcvm.2023.1162205 PMC10151546 37144056

[22] Campuzano O, Alcalde M, Iglesias A, Barahona-Dussault C, Sarquella-Brugada G, Benito B, et al. Arrhythmogenic right ventricular cardiomyopathy: severe structural alterations are associated with inflammation. J Clin Pathol. 2012;65(12):1077–83. 22944624 10.1136/jclinpath-2012-201022

[23] Lubos N, van der Gaag S, Gerçek M, Kant S, Leube RE, Krusche CA. Inflammation shapes pathogenesis of murine arrhythmogenic cardiomyopathy. Basic Res Cardiol. 2020;115(4):42. 32529556 10.1007/s00395-020-0803-5 PMC7289786

[24] Valente M, Calabrese F, Thiene G, Angelini A, Basso C, Nava A, et al. In vivo evidence of apoptosis in arrhythmogenic right ventricular cardiomyopathy. Am J Pathol. 1998;152(2):479–84. 9466574 PMC1857974

[25] Meraviglia V, Alcalde M, Campuzano O, Bellin M. Inflammation in the Pathogenesis of Arrhythmogenic Cardiomyopathy: Secondary Event or Active Driver? Front Cardiovasc Med [Internet]. 2021 Dec 20 [cited 2024 Aug 31];8. Available from: https://www.frontiersin.org/journals/cardiovascular-medicine/articles/10.3389/fcvm.2021.784715/full 10.3389/fcvm.2021.784715 PMC8720743 34988129

[26] Kontorovich AR, Patel N, Moscati A, Richter F, Peter I, Purevjav E, et al. Myopathic cardiac genotypes increase risk for myocarditis. JACC Basic Transl Sci. 2021;6(7):584–92. 34368507 10.1016/j.jacbts.2021.06.001 PMC8326270

[27] Chelko SP, Asimaki A, Lowenthal J, Bueno-Beti C, Bedja D, Scalco A, et al. Therapeutic modulation of the immune response in arrhythmogenic cardiomyopathy. Circulation. 2019;140(18):1491–505. 31533459 10.1161/CIRCULATIONAHA.119.040676 PMC6817418

[28] Corrado D, Basso C, Thiene G, McKenna WJ, Davies MJ, Fontaliran F, et al. Spectrum of clinicopathologic manifestations of arrhythmogenic right ventricular cardiomyopathy/dysplasia: a multicenter study. J Am Coll Cardiol. 1997;30(6):1512–20. 9362410 10.1016/s0735-1097(97)00332-x

[29] Basso C, Thiene G, Corrado D, Angelini A, Nava A, Valente M. Arrhythmogenic right ventricular cardiomyopathy. Circulation. 1996;94(5):983–91. 8790036 10.1161/01.cir.94.5.983

[30] Asatryan B, Asimaki A, Landstrom AP, Khanji MY, Odening KE, Cooper LT, et al. Inflammation and immune response in arrhythmogenic cardiomyopathy: state-of-the-art review. Circulation. 2021;144(20):1646–55. 34780255 10.1161/CIRCULATIONAHA.121.055890 PMC9034711

[31] Mavroidis M, Davos CH, Psarras S, Varela AC, Athanasiadis N, Katsimpoulas M, et al. Complement system modulation as a target for treatment of arrhythmogenic cardiomyopathy. Basic Res Cardiol. 2015;110(3):27. 25851234 10.1007/s00395-015-0485-6

[32] Chen L, Yi M, Song J, Hu S. Complement system is highly activated and potentially acts as a biomarker for patients with arvc. J Am Coll Cardiol. 2018;71(11_Supplement):A745–A745.

[33] Chelko SP, Penna VR, Engel M, Shiel EA, Centner AM, Farra W, et al. NFĸB signaling drives myocardial injury via CCR2^+^ macrophages in a preclinical model of arrhythmogenic cardiomyopathy. J Clin Invest [Internet]. 2024 Jul 5 [cited 2024 Aug 31];134(10). Available from: https://www.jci.org/articles/view/172014 10.1172/JCI172014 PMC11093597 38564300

[34] Hawthorne RN, Blazeski A, Lowenthal J, Kannan S, Teuben R, DiSilvestre D, et al. Altered electrical, biomolecular, and immunologic phenotypes in a novel patient-derived stem cell model of desmoglein-2 Mutant ARVC. J Clin Med. 2021;10(14):3061. 34300226 10.3390/jcm10143061 PMC8306340

[35] Bowles NE, Ni J, Marcus F, Towbin JA. The detection of cardiotropic viruses in the myocardium of patients with arrhythmogenic right ventricular dysplasia/cardiomyopathy. J Am Coll Cardiol. 2002;39(5):892–5. 11869858 10.1016/s0735-1097(02)01688-1

[36] Calabrese F, Basso C, Carturan E, Valente M, Thiene G. Arrhythmogenic right ventricular cardiomyopathy/dysplasia: is there a role for viruses? Cardiovasc Pathol. 2006;15(1):11–7. 16414451 10.1016/j.carpath.2005.10.004

[37] Calabrese F, Angelini A, Thiene G, Basso C, Nava A, Valente M. No detection of enteroviral genome in the myocardium of patients with arrhythmogenic right ventricular cardiomyopathy. J Clin Pathol. 2000;53(5):382–7. 10889821 10.1136/jcp.53.5.382 PMC1731194

[38] Chatterjee D, Fatah M, Akdis D, Spears DA, Koopmann TT, Mittal K, et al. An autoantibody identifies arrhythmogenic right ventricular cardiomyopathy and participates in its pathogenesis. Eur Heart J. 2018;39(44):3932–44. 30239670 10.1093/eurheartj/ehy567 PMC6247665

[39] Caforio ALP, Re F, Avella A, Marcolongo R, Baratta P, Seguso M, et al. Evidence from family studies for autoimmunity in arrhythmogenic right ventricular cardiomyopathy: associations of circulating anti-heart and anti-intercalated disk autoantibodies with disease severity and family history. Circulation. 2020;141(15):1238–48. 32114801 10.1161/CIRCULATIONAHA.119.043931

[40] Catapano D, Tontodonato M, D’Elia S, Pezzullo E, Ciaramella F, Vettori S, et al. Fulminant myocarditis unmasking adult-onset still’s disease and desmoplakin truncation. Circ Cardiovasc Imaging. 2023;16(8):e015001. 37283033 10.1161/CIRCIMAGING.122.015001

[41] Gonano N, Nuzzi V, Pavan D, Piazza R, Pecoraro R, Altinier A, et al. ‘Hot phase’ non-dilated left ventricular cardiomyopathy with atypical onset and recurrence: a case report. ESC Heart Fail [Internet]. [cited 2024 Aug 31];n/a(n/a). Available from: https://onlinelibrary.wiley.com/doi/abs/10.1002/ehf2.14822 10.1002/ehf2.14822 PMC11631281 39001591

[42] Guan F, Wolber T, Saguner AM, Medeiros A, Müggler O, Berger F, et al. A desmoplakin variant associated with isolated arrhythmogenic left ventricular cardiomyopathy with rapid monomorphic ventricular tachycardia at first presentation. Hear Case Rep. 2023;9(6):406–9. 10.1016/j.hrcr.2023.03.017 PMC10285122 37361972

[43] Bauce B, Basso C, Rampazzo A, Beffagna G, Daliento L, Frigo G, et al. Clinical profile of four families with arrhythmogenic right ventricular cardiomyopathy caused by dominant desmoplakin mutations. Eur Heart J. 2005;26(16):1666–75. 15941723 10.1093/eurheartj/ehi341

[44] Piriou N, Marteau L, Kyndt F, Serfaty JM, Toquet C, Le Gloan L, et al. Familial screening in case of acute myocarditis reveals inherited arrhythmogenic left ventricular cardiomyopathies. ESC Heart Fail. 2020;7(4):1520–33. 32356610 10.1002/ehf2.12686 PMC7373927

[45] Engel M, Shiel EA, Chelko SP. Basic and translational mechanisms in inflammatory arrhythmogenic cardiomyopathy. Int J Cardiol. 2024;15(397):131602. 10.1016/j.ijcard.2023.131602 37979796

[46] Akdis D, Chen L, Saguner AM, Zhang N, Gawinecka J, Saleh L, et al. Novel plasma biomarkers predicting biventricular involvement in arrhythmogenic right ventricular cardiomyopathy. Am Heart J. 2022;244:66–76. 34756894 10.1016/j.ahj.2021.10.187

[47] Chimenti C, Magnocavallo M, Vetta G, Alfarano M, Manguso G, Ajmone F, et al. The role of MicroRNA in the myocarditis: a small actor for a great role. Curr Cardiol Rep. 2023;25(7):641–8. 37269474 10.1007/s11886-023-01888-5 PMC10307691

[48] Protonotarios A, Wicks E, Ashworth M, Stephenson E, Guttmann O, Savvatis K, et al. Prevalence of 18F-fluorodeoxyglucose positron emission tomography abnormalities in patients with arrhythmogenic right ventricular cardiomyopathy. Int J Cardiol. 2019;1(284):99–104. 10.1016/j.ijcard.2018.10.083 30409737

[49] Peretto G, Busnardo E, Ferro P, Palmisano A, Vignale D, Esposito A, et al. Clinical applications of FDG-PET scan in arrhythmic myocarditis. JACC Cardiovasc Imaging. 2022;15(10):1771–80. 36202457 10.1016/j.jcmg.2022.02.029

[50] Lal M, Chen C, Newsome B, Masha L, Camacho SA, Masri A, et al. Genetic cardiomyopathy masquerading as cardiac sarcoidosis. J Am Coll Cardiol. 2023;81(1):100–2. 36599603 10.1016/j.jacc.2022.10.021

[51] Corrado D, Basso C, Judge DP. Arrhythmogenic cardiomyopathy. Circ Res. 2017;121(7):784–802. 28912183 10.1161/CIRCRESAHA.117.309345

[52] Bassetto G, Merlo M, Dal Ferro M, Setti M, Paldino A, Collesi C, et al. Apoptosis, a useful marker in the management of hot-phase cardiomyopathy? Eur J Heart Fail. 2024;26(3):590–7. 38414301 10.1002/ejhf.3173

[53] Casella M, Dello Russo A, Bergonti M, Catto V, Conte E, Sommariva E, et al. Diagnostic yield of electroanatomic voltage mapping in guiding endomyocardial biopsies. Circulation. 2020;142(13):1249–60. 32791857 10.1161/CIRCULATIONAHA.120.046900

[54] Arbelo E, Protonotarios A, Gimeno JR, Arbustini E, Barriales-Villa R, Basso C, et al. 2023 ESC guidelines for the management of cardiomyopathies: Developed by the task force on the management of cardiomyopathies of the European Society of Cardiology (ESC). Eur Heart J. 2023;44(37):3503–626. 37622657 10.1093/eurheartj/ehad194

[55] McDonagh TA, Metra M, Adamo M, Gardner RS, Baumbach A, Böhm M, et al. 2021 ESC Guidelines for the diagnosis and treatment of acute and chronic heart failure: Developed by the Task Force for the diagnosis and treatment of acute and chronic heart failure of the European Society of Cardiology (ESC) With the special contribution of the Heart Failure Association (HFA) of the ESC. Eur Heart J. 2021;42(36):3599–726. 34447992 10.1093/eurheartj/ehab368

[56] Chimenti C, Russo MA, Frustaci A. Immunosuppressive therapy in virus-negative inflammatory cardiomyopathy: 20-year follow-up of the TIMIC trial. Eur Heart J. 2022;43(36):3463–73. 35831932 10.1093/eurheartj/ehac348 PMC9492235

[57] Peretto G, De LG, Villatore A, Di RC, Sala S, Palmisano A, et al. Multimodal detection and targeting of biopsy-proven myocardial inflammation in genetic cardiomyopathies. JACC Basic Transl Sci. 2023;8(7):755–65. 37547072 10.1016/j.jacbts.2023.02.018 PMC10401291

[58] Caforio ALP, Giordani AS, Baritussio A, Marcolongo D, Vicenzetto C, Tarantini G, et al. Long-term efficacy and safety of tailored immunosuppressive therapy in immune-mediated biopsy-proven myocarditis: A propensity-weighted study. Eur J Heart Fail. 2024;26(5):1175–85. 38629741 10.1002/ejhf.3220

[59] Chelko SP, Asimaki A, Andersen P, Bedja D, Amat-Alarcon N, DeMazumder D, et al. Central role for GSK3β in the pathogenesis of arrhythmogenic cardiomyopathy. JCI Insight [Internet]. 2016 Apr 21 [cited 2024 Aug 31];1(5). Available from: https://insight.jci.org/articles/view/85923 10.1172/jci.insight.85923 PMC4861310 27170944

[60] Asimaki A, Kapoor S, Plovie E, Karin Arndt A, Adams E, Liu Z, et al. Identification of a New Modulator of the Intercalated Disc in a Zebrafish Model of Arrhythmogenic Cardiomyopathy. Sci Transl Med. 2014;6(240):240ra74-240ra74. 24920660 10.1126/scitranslmed.3008008 PMC4471875

[61] Chelko SP, Penna V, Engel M, Landim-Vieira M, Cannon EN, Lavine K, et al. Mechanisms of Innate Immune Injury in Arrhythmogenic Cardiomyopathy [Internet]. bioRxiv; 2023 [cited 2024 Aug 31]. p. 2023.07.12.548682. Available from: https://www.biorxiv.org/content/10.1101/2023.07.12.548682v1

[62] Mesquita T, Cingolani E. Targeting arrhythmogenic macrophages: lessons learned from arrhythmogenic cardiomyopathy. J Clin Invest [Internet]. 2024 May 15 [cited 2024 Aug 18];134(10). Available from: https://www.jci.org/articles/view/180482 10.1172/JCI180482 PMC11093592 38747296

[63] ctv.veeva.com [Internet]. [cited 2024 Aug 31]. CMP-MYTHiC Trial and Registry - CardioMyoPathy With MYocarditis THerapy With Colchicine. Available from: https://ctv.veeva.com/study/cmp-mythic-trial-and-registry-cardiomyopathy-with-myocarditis-therapy-with-colchicine

[64] Wang W, Orgeron G, Tichnell C, Murray B, Crosson J, Monfredi O, et al. Impact of Exercise Restriction on Arrhythmic Risk Among Patients With Arrhythmogenic Right Ventricular Cardiomyopathy. J Am Heart Assoc. 2018;7(12):e008843. 29909402 10.1161/JAHA.118.008843 PMC6220537

[65] Gasperetti A, Carrick R, Protonotarios A, Laredo M, Van der SI, Syrris P, et al. Long-term arrhythmic follow-up and risk stratification of patients with desmoplakin-associated arrhythmogenic right ventricular cardiomyopathy. JACC Adv. 2024;3(3):100832. 38938828 10.1016/j.jacadv.2024.100832 PMC11198598

[66] Lopez-Ayala JM, Pastor-Quirante F, Gonzalez-Carrillo J, Lopez-Cuenca D, Sanchez-Munoz JJ, Oliva-Sandoval MJ, et al. Genetics of myocarditis in arrhythmogenic right ventricular dysplasia. Heart Rhythm. 2015;12(4):766–73. 25616123 10.1016/j.hrthm.2015.01.001

